# Integrative analysis of metabolome, proteome, and transcriptome for identifying genes influencing total lignin content in *Populus trichocarpa*

**DOI:** 10.3389/fpls.2023.1244020

**Published:** 2023-09-13

**Authors:** Jia Zhao, Kairui Chao, Achuan Wang

**Affiliations:** ^1^ College of Computer and Control Engineering, Northeast Forestry University, Harbin, China; ^2^ College of Forestry, Inner Mongolia Agricultural University, Hohhot, China

**Keywords:** total lignin content, multi-omics, WGCNA, differential genes, enrichment analysis, co-expression network, correlation analysis

## Abstract

Lignin, a component of plant cell walls, possesses significant research potential as a renewable energy source to replace carbon-based products and as a notable pollutant in papermaking processes. The monolignol biosynthetic pathway has been elucidated and it is known that not all monolignol genes influence the total lignin content. However, it remains unclear which monolignol genes are more closely related to the total lignin content and which potential genes influence the total lignin content. In this study, we present a combination of t-test, differential gene expression analysis, correlation analysis, and weighted gene co-expression network analysis to identify genes that regulate the total lignin content by utilizing multi-omics data from transgenic knockdowns of the monolignol genes that includes data related to the transcriptome, proteome, and total lignin content. Firstly, it was discovered that enzymes from the *PtrPAL*, *Ptr4CL*, *PtrC3H*, and *PtrC4H* gene families are more strongly correlated with the total lignin content. Additionally, the co-downregulation of three genes, *PtrC3H3*, *PtrC4H1*, and *PtrC4H2*, had the greatest impact on the total lignin content. Secondly, GO and KEGG analysis of lignin-related modules revealed that the total lignin content is not only influenced by monolignol genes, but also closely related to genes involved in the “glutathione metabolic process”, “cellular modified amino acid metabolic process” and “carbohydrate catabolic process” pathways. Finally, the cinnamyl alcohol dehydrogenase genes *CAD1*, *CADL3*, and *CADL8* emerged as potential contributors to total lignin content. The genes *HYR1* (UDP-glycosyltransferase superfamily protein) and *UGT71B1* (UDP-glucosyltransferase), exhibiting a close relationship with coumarin, have the potential to influence total lignin content by regulating coumarin metabolism. Additionally, the monolignol genes *PtrC3H3*, *PtrC4H1*, and *PtrC4H2*, which belong to the cytochrome P450 genes, may have a significant impact on the total lignin content. Overall, this study establishes connections between gene expression levels and total lignin content, effectively identifying genes that have a significant impact on total lignin content and offering novel perspectives for future lignin research endeavours.

## 1 Introduction

Lignin is the second most abundant complex aromatic polymer in Plantae, following cellulose, and is predominantly found in the woody tissues of plants. As a major component of plant cell walls, lignin plays a crucial role in preventing the invasion of various plant pathogens, contributing significantly to plant defence against external abiotic stresses (Boerjan et al., 2003; Vanholme et al., 2010). Moreover, wood holds promise as a renewable energy source due to its ability to release more heat when used as fuel, offering significant potential for replacing other carbon-based products (de Vries et al., 2021). The monolignol biosynthetic pathway begins with phenylalanine, a product of the phenylalanine pathway. Phenylalanine is catalytically converted into cinnamic acid by phenylalanine ammonia-lyase (PAL). Cinnamic acid is then hydroxylated by cinnamate 4-hydroxylase (C4H) to produce p-coumaric acid. Subsequently, p-coumaric acid is catalysed by 4-coumarate-CoA ligase (4CL) to form p-coumaroyl-CoA, which enters the specific monolignol biosynthesis pathway. Various enzymes, including cinnamate 3-hydroxylase (C3H), hydroxycinnamoyl transferase (HCT), and cinnamoyl-CoA reductase (CCR), catalyse the formation of monolignols. These monolignols are composed of three major subunits: hydroxyphenyl (H), guaiacyl (G), and syringyl (S) monolignols. Finally, through the random combination of various monolignols, polymerization occurs, resulting in lignin with complex structures (Umezawa, 2010; Vanholme et al., 2019; Zhang et al., 2020). Research has shown that laccase (LAC) and peroxidase (POD) play important roles in the polymerization of monolignols into lignin with complex structures (Long et al., 2021; Wen et al., 2022).

**Figure 1:**
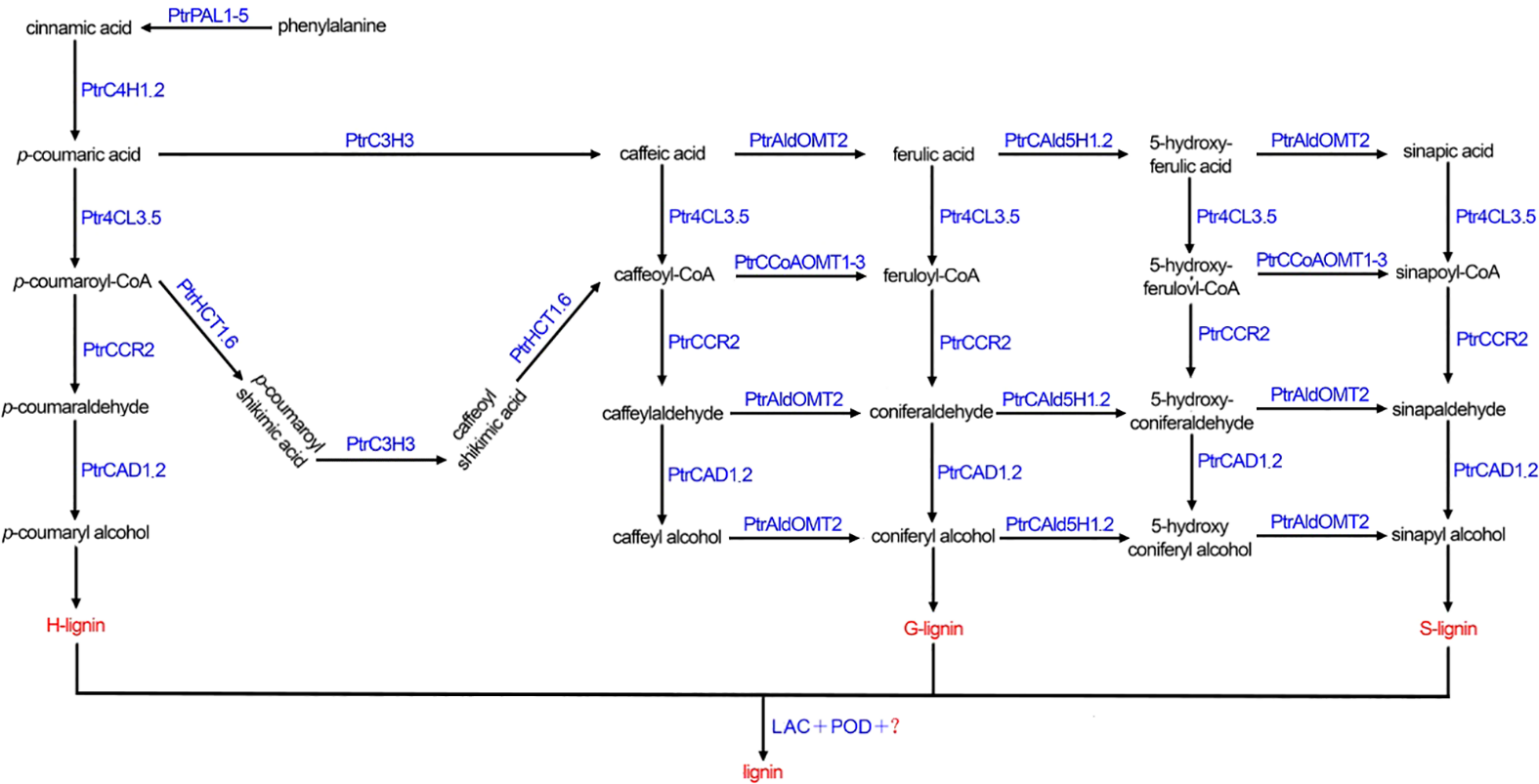
The monolignol biosynthetic pathway in *Populus trichocarpa*. PtrPAL1-5: *PtrPAL1*, *PtrPAL2*, *PtrPAL3*, *PtrPAL4*, *PtrPAL5*; PtrC4H1.2: *PtrC4H1*, *PtrC4H2*; Ptr4CL3.5: *Ptr4CL3*, *Ptr4CL5*; PtrCAD1.2: *PtrCAD1*, *PtrCAD2*; PtrHCT1.6: *PtrHCT1*, *PtrHCT6*; PtrCAld5H1.2: *PtrCAld5H1*, *PtrCAld5H2*; PtrCCoAOMT1-3: *PtrCCoAOMT1*, *PtrCCoAOMT2*, *PtrCCoAOMT3*; LAC, laccase; POD, peroxidase.

Poplar is an important tree species in China, that is known for its fast growth and ease of asexual reproduction, which facilitates the smooth progress of genetic engineering technology. As a model species, the complete genome of *Populus trichocarpa* was the first woody plant to be sequenced (Tuskan et al., 2006). After four years, Shi et al. (Shi et al., 2010) comprehensively identified monolignol genes in *P. trichocarpa* and identified 23 wood-specific enzymes involved in ten monolignol gene families, including PAL, 4CL, C3H, C4H, HCT, CCR, CAD (cinnamyl alcohol dehydrogenase), CCoAOMT (caffeoyl-CoA O-methyltransferase), COMT (Catechol-O-methyltransferase) and CAld5H (coniferaldehyde-5-hydroxylase). The monolignol biosynthetic pathway and the intermediate metabolites in *P. trichocarpa* are depicted in **Figure 1**.

Genetic modifications are valuable methods for regulating metabolic pathways. These modifications alter the expression levels of genes, resulting in changes in the enzyme content that can be used to catalyse key pathway reactions, thereby achieving the regulation of lignin content. In genetic engineering studies of *P. trichocarpa*, it was found that the genes *Ptr4CL*, *PtrHCL*, *PtrCCR*, and *PtrCAD* not only reduced the total lignin content but also influenced the lignin composition. On the other hand, the genes *PtrC3H*, *PtrC4H*, *PtrPAL*, and *PtrCOMT* solely led to a reduction in total lignin content. The gene *PtrCAld5H* increased the total lignin content and influenced the composition of lignin. However, the gene *PtrCCoAOMT* had no effect on either the total lignin content or composition (Peng et al., 2016; Van Acker et al., 2017; Wang et al., 2018; De Meester et al., 2020; Tsai et al., 2020). In addition, Kim et al. found that the simultaneous knockdown of the three genes *PtrC4H1*, *PtrC4H2*, and *PtrC3H3* significantly reduced the total lignin content in *P. trichocarpa* (Kim et al., 2020). However, there are certain limitations to studying the relationship between gene editing of monolignol genes and lignin content using genetic engineering techniques. The focus of such studies is more on investigating the regulation of the monolignol biosynthetic pathway rather than uncovering the specific genes that influence lignin content. Additionally, editing the same monolignol genes in different species may lead to varying effects on lignin content. Therefore, it is crucial to employ more appropriate analytical methods and explore the genes that impact lignin content in a specific species.

Gene co-expression networks (GCNs) have been widely used for gene identification and weighted gene co-expression network analysis (WGCNA) is the most representative GCN (Zhang and Horvath, 2005). WGCNA entails a clustering analysis of gene expression levels from microarray or RNA-seq data, representing relationships between gene nodes through continuous variable edge weights. Notably, WGCNA addresses the issue of “one-size-fits-all” in traditional GCN construction by using a unique dynamic cut method. Furthermore, highly correlated gene sets (gene modules) obtained through clustering, which have similar functions, can be associated with sample traits by calculating correlation coefficients. Importantly, WGCNA allows for the simultaneous analysis of gene modules and multiple traits related to biological relevance, enabling the identification of biologically significant genes and establishing co-expression relationships among genes within modules and their associations with relevant traits (Wu C. et al., 2021; Kondoh et al., 2022; Petrosyan et al., 2023). Rao et al. (Rao et al., 2019) and Hong et al. (Hong et al., 2021) applied gene co-expression network analysis methods to explore genes and transcription factors related to the monolignol biosynthetic pathway in *P. trichocarpa*. However, their studies focused on establishing network relationships between genes without specifically analysing the impact of genes on lignin content. Therefore, it is of great importance to construct a co-expression network of genes closely associated with total lignin content. This can be achieved by utilizing data from transcriptome, proteome, and total lignin content measurements following the knockdown of multiple monolignol genes. Such an approach can effectively identify genes that significantly influence the total lignin content.

In this paper, we focus on the genes that influence total lignin content in *P. trichocarpa*. To achieve this, we conducted a comprehensive analysis of multi-omics data, which includes transcriptome, proteome, and total lignin content, in both transgenic lines with knockdown genes of ten monolignol gene families and control lines. We present a combination of t-tests, differential gene expression analysis, correlation analysis, and WGCNA to identify genes that influence the total lignin content. Specifically, we started by using t-tests to identify 15 transgenic lines with significant differences. Then, we performed differential gene expression analysis on each of these 15 groups individually and selected differentially expressed genes with at least one intersecting gene among the 15 groups, resulting in a total of 5894 genes and 139 samples. To identify important gene modules closely related to lignin, we constructed tightly interconnected gene modules based on the theory that genes with similar functions exhibit similar expression patterns. We also incorporated the absolute protein abundance of the monolignol biosynthetic enzymes and total lignin content data in this process. Next, we performed GO and KEGG enrichment analyses to investigate the functions of genes within specific modules and identify the biological pathways in which the genes may be involved, and have an impact on total lignin content. Furthermore, we conducted correlation analysis between the expression levels of genes in the modules most closely associated with total lignin content and the actual lignin content. Using the 15 genes that showed a strong correlation with total lignin content as the core, we constructed a weighted gene co-expression network to explore the genes influencing total lignin content. This research provides a solid foundation for understanding lignin synthesis and degradation processes, and it offers valuable insights for the development and utilization of lignin.

## 2 Materials and methods

### 2.1 Data availability

Wang et al. performed a series of systematic experimental knockdowns of monolignol genes, and the absolute abundances of the monolignol transcripts and proteins were measured using RNA-seq and protein cleavage isotope dilution mass spectrometry (PC-IDMS), respectively. The lignin content was determined following the Klason procedure in *P. trichocarpa* (Wang et al., 2018). The transcriptomics data is available under GEO accession number GSE78953 [https://www.ncbi.nlm.nih.gov/geo/query/acc.cgi?acc=GSE78953], and the proteomics dataset and total lignin content are accessible on CyVerse [https://datacommons.cyverse.org/browse/iplant/home/shared/LigninSystemsDB].

We analysed the transcriptome, proteome (20 pathway enzymes), and total lignin content data of both transgenic lines with multiple gene knockdowns of ten monolignol gene families (*PtrPAL*, *PtrC3H*, *PtrC4H*, *PtrCAD*, *PtrCCR*, *PtrHCT*, *Ptr4CL*, *PtrCAld5H*, *PtrCOMT*, and *PtrCCoAOMT*), and corresponding control lines. The annotation information of genes was obtained from two databases, JGI [https://jgi.doe.gov/] and NCBI [https://www.ncbi.nlm.nih.gov/].

### 2.2 T-test and differential gene analysis

**Figure 2:**
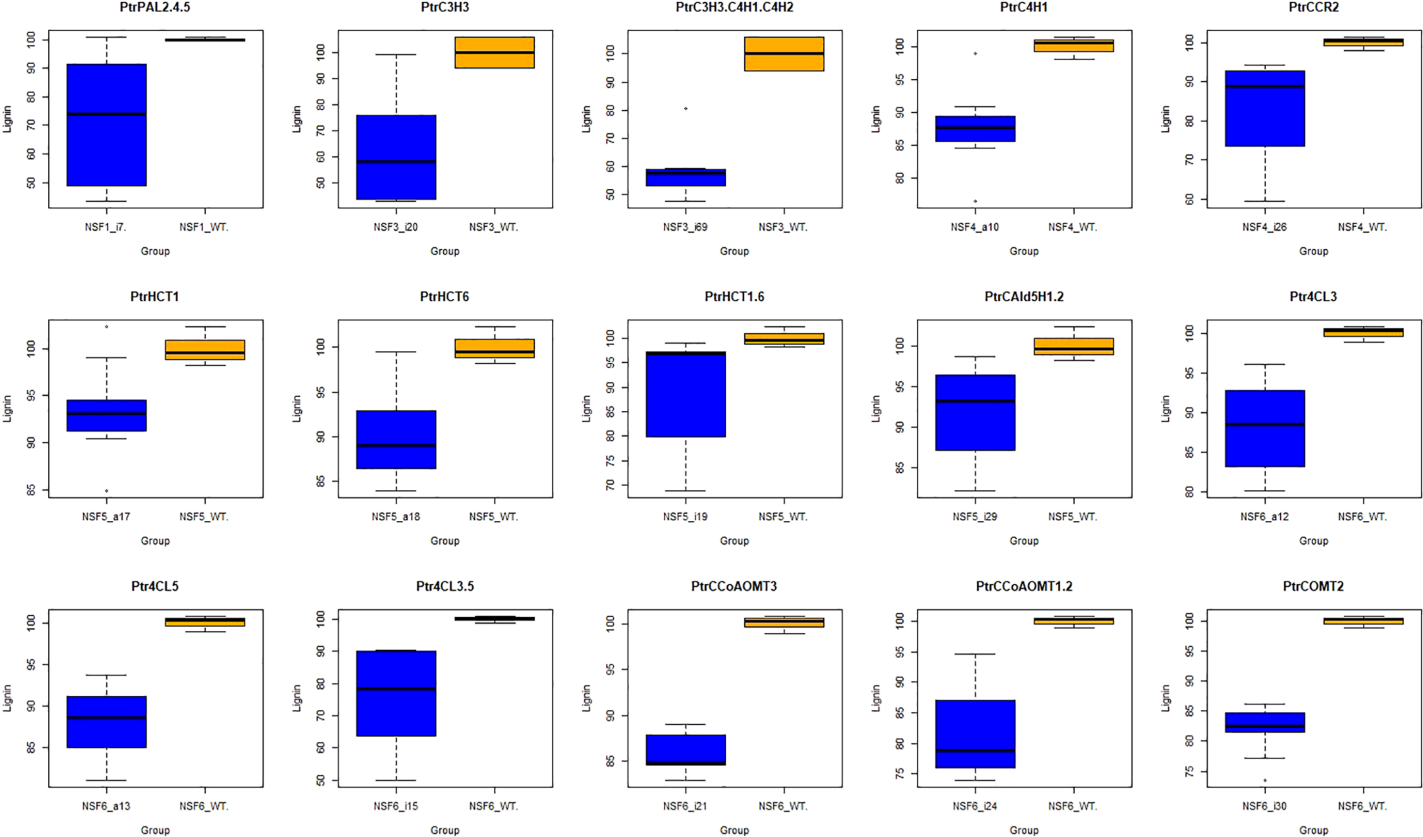
Boxplot of 15 groups. The blue box represent traitements, and the orange box represent corresponding controls. Target genes of 15 traitement groups are listed on the top of the panel. PtrPAL2.4.5: the co-downregulation of *PtrPAL2*, *PtrPAL4*, and *PtrPAL5*; PtrC3H3.C4H1.C4H2: the co-downregulation of *PtrC3H3*, *PtrC4H1*, and *PtrC4H2*; PtrHCT1.6: the co-downregulation of *PtrHCT1* and *PtrHCT6*; PtrCAld5H1.2: the co-downregulation of *PtrCAld5H1* and *PtrCAld5H2*; the co-downregulation of Ptr4CL3.5: *Ptr4CL3* and *Ptr4CL5*; PtrCCoAOMT1.2: the co-downregulation of *PtrCCoAOMT1* and *PtrCCoAOMT2*.

**Figure 3:**
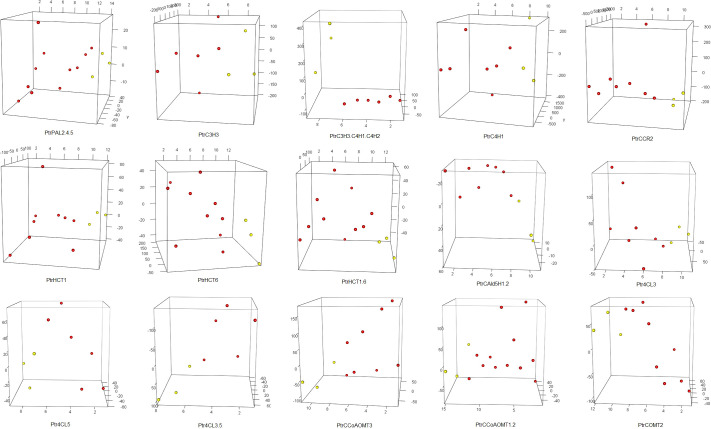
PCA of 15 groups. The red dots represent traitement samples, and the yellow dots represent corresponding control samples. Target genes of 15 traitement groups are listed on the bottom of the panel. The abbreviations for the genes in the treatment group are the same as the caption in **Figure 2**.

**Figure 4:**
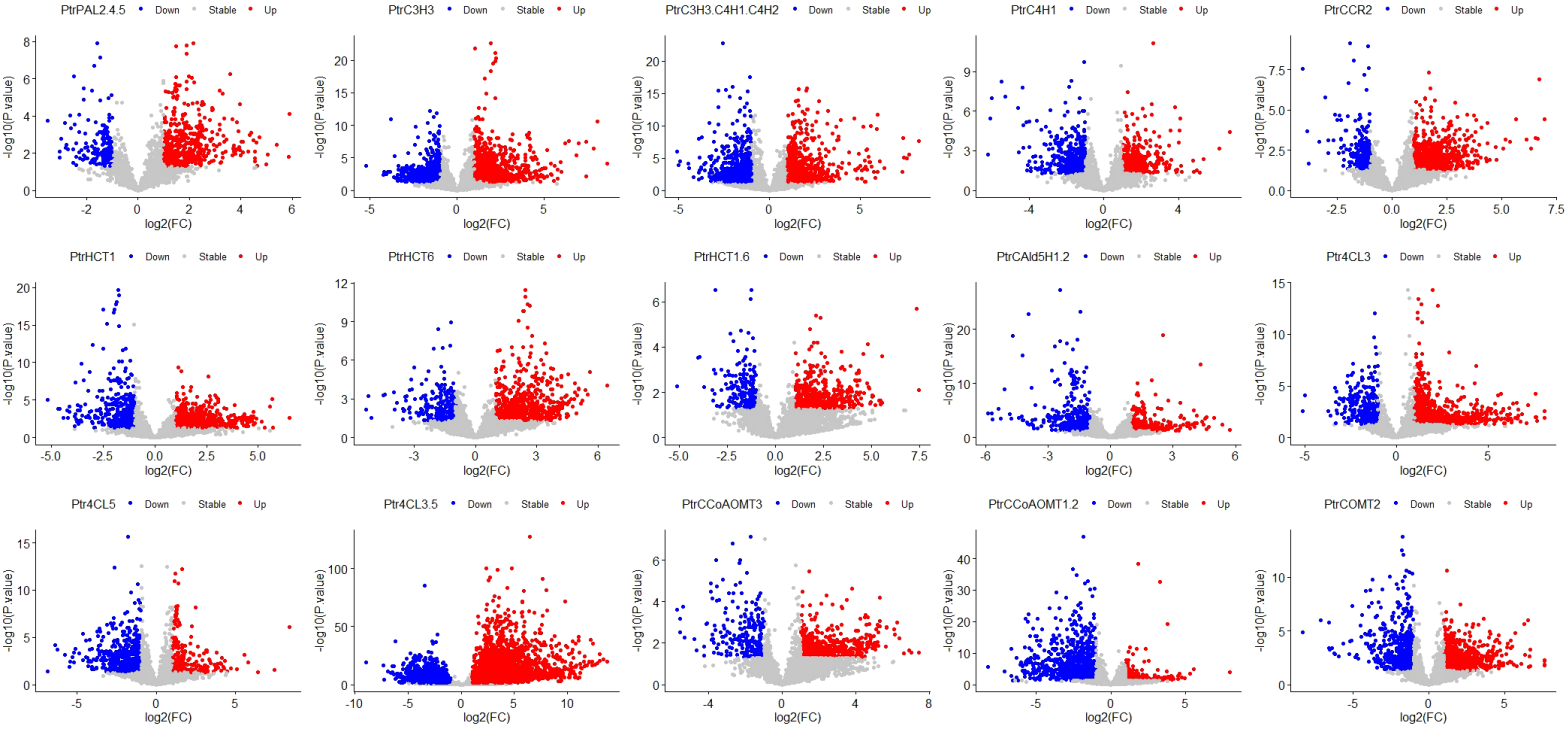
Volcano Plot of 15 groups. The red dots represent significantly upregulated genes, the grey dots represent stable genes, and the blue dots represent significantly downregulated genes. Target genes of 15 traitement groups are listed on the top of the panel. The abbreviations for the genes in the treatment group are the same as the caption in **Figure 2**.

Firstly, we conducted t-tests using the R package t.test on the total lignin content of all different knockdown transgenic and control lines (**Table S1**). As a result, we identified 15 groups of transgenic lines that displayed significant differences (P<0.05) in total lignin content. We visualized these results using boxplots (**Figure 2**). These transgenic lines were obtained through various treatments involving the knockdown of specific genes (*PtrPAL2.4.5*, *PtrC3H3*, *PtrC3H3.C4H1.2*, *PtrC4H1*, *PtrHCT1*, *PtrHCT6*, *PtrHCT1,6*, *PtrCAld5H1,2*, *PtrCCR2*, *PtrCCoAOMT1.2*, *PtrCCoAOMT3*, *PtrCOMT2*, *Ptr4CL3*, *Ptr4CL5*, and *Ptr4CL3.5*). Subsequently, we performed average clustering analysis on the 15 groups of transgenic and control lines, eliminating obvious outliers, and generated PCA plots (**Figure 3**) and volcano plots (**Figure 4**).

Furthermore, we performed differential gene analysis using the R package DESeq2 on the data of the 15 groups of transgenic and control lines, with filtering criteria set as log2FC > 1 and Padj< 0.05. To ensure a more accurate identification of genes regulating total lignin content, we selected the differential genes that had at least one intersection among the 15 groups for further analysis. After these steps, a total of 5894 differentially expressed genes from 139 samples were used for WGCNA.

### 2.3 Data preprocessing

Data preprocessing plays a vital role before conducting WGCNA, as it guarantees data consistency, completeness, and suitability for analysis, ultimately ensuring data quality and enhancing the accuracy and reliability of the results. The transcriptome, proteome, and total lignin data cannot be directly utilized in WGCNA; instead, they necessitate data preprocessing steps, including data filtering, normalization, and standardization. These steps are essential to prepare the data in a suitable format for meaningful and effective network analysis.

#### 2.3.1 Data filtering

The RNA-seq results indicate that not all genes are expressed, and the expression levels of each gene can vary greatly under different biological processes, sampling times, and tissue locations. When analysing gene expression data, genes with low expression levels have lower reliability and need to be filtered out to ensure the reliability of data analysis. To achieve this, we transformed the read counts (reads) aligned to the reference gene fragments into CPM (counts-per-million threshold). Subsequently, we excluded genes with CPM values less than 0.5 in all three sample replicates to effectively remove genes with low expression levels. For this filtering process, we utilized the “cpm” function available in the R package edgeR (Robinson et al., 2010). The formula (1) for calculating CPM is as follows:


(1) $$\text{CPM}=\frac{C}{N}10^{6}$$

Where *C* represents the reads aligned to a specific gene, and *N* represents the total number of reads aligned to all genes in the sample.

#### 2.3.2 Data normalization

Due to limited experimental space, all experimental samples were obtained in six different batches, and each batch of transgenic lines had corresponding controls. We normalized the counts of genes to their corresponding wild-type data using the ratio-based (arithmetic mean as reference, Ratio-A) approach (Luo et al., 2010; Li and Zhao, 2019). The formula (2) is as follows:


(2) $$X_{ij}^{'}=X_{ij}-\frac{1}{n}\sum_{l=1}^{n}X_{il}^{r}$$

where *r* is the reference batch, $X_{ij}^{'}\;$ represents the adjusted gene expression value of the *i*-th gene in the *j*-th sample, $X_{ij}$ represents the original gene expression value of the *i*-th gene in the *j*-th sample, *n* is the number of control samples in the reference batch, and $X_{il}^{r}$ represents the gene expression value of the *i*-th gene in the *l*-th sample of the reference batch. Additionally, since WGCNA requires input data to be greater than or equal to 0, we added the absolute value of the smallest negative number to all corrected counts in all batches.

#### 2.3.3 Data standardization

Gene expression levels are determined by randomly sampling gene fragments, leading to a higher likelihood of sampling long sequence genes compared to short ones. Additionally, the sequencing depth can impact the number of reads aligned to each gene. Thus, relying solely on raw reads is inadequate to accurately measure gene expression levels. To address this issue, we adopted TPM (transcripts per million) normalization to convert the reads into the number of reads per kilobase per million mapped reads, offering a more reliable measure of gene expression for analysis. The formula (3) for calculating TPM is as follows:


(3) $$\text{TPM}=\frac{Ri}{(sum\frac{Ri}{li})*li}10^{6}$$

Where *Ri* represents the number of reads for the *i*-th gene, *li* represents the length of the *i*-th gene (in kilobases), and *sum* $\frac{Ri}{li}$ is the sum of ratios between the reads of the *i*-th gene and its corresponding gene length. The multiplication by 10^6^ is to convert it into the number of reads per million mapped reads.

### 2.4 Weighted gene co-expression network analysis

In WGCNA, gene expression data are utilized to calculate the Pearson correlation coefficient between genes, resulting in a gene co-expression similarity matrix through correlation analysis. Traditionally, a hard threshold is employed to determine whether genes are correlated or not. This means that values above the threshold indicate correlation, while values below it indicate no correlation. However, this approach may lead to some information loss. To address this, WGCNA adopts a soft thresholding approach, assuming that there is a correlation between all genes. This approach transforms the gene expression similarity matrix into an adjacency matrix and subsequently into a topological overlap matrix, which measures the interconnectedness among genes. By doing so, the method considers not only the pairwise correlation between two genes but also their correlation with other genes in the network. To identify gene modules, the dynamic tree cut algorithm (Langfelder et al., 2008) are applied. This algorithm mimics the characteristics of real biological networks, providing biologically meaningful results. Overall, the soft thresholding approach and dynamic tree cut algorithm in WGCNA allow for a more comprehensive analysis, capturing the complexity of gene interactions and facilitating the identification of functionally related gene modules.

#### 2.4.1 Construction of gene modules

After preprocessing the gene expression data, the genes were further subjected to detection and filtering using the goodSamplesGenes functions available in the WGCNA package within the R programming environment. The selected genes that met the criteria were used to construct a weighted gene co-expression network using the WGCNA package in R 4.2.2 software. For the specific methods and principles employed in this process, please refer to the research conducted by Zhang B et al. (Zhang and Horvath, 2005).

#### 2.4.2 GO and KEGG enrichment analyses

We conducted functional annotation of genes within specific modules using two important databases: the Gene Ontology (GO) and the Kyoto Encyclopedia of Genes and Genomes (KEGG). The GO (Zhao et al., 2020) database [http://geneontology.org/] offers a standardized language to define and describe gene and gene product functions across various biological species. GO terms classify gene functions into three categories: Molecular Function (MF), Biological Process (BP), and Cellular Component (CC). These terms enable researchers to predict the functions of genes or proteins in the same or different species based on the known functions of genes or proteins stored in the database. On the other hand, the KEGG (Chen et al., 2020) database [https://www.kegg.jp/] serves as a knowledge repository for systematic analysis of gene functions. It integrates genomic, chemical, and systemic functional information and contains a vast amount of genomic sequence data, pathway information related to metabolism, regulation, and signal transduction, as well as information on chemical compounds, enzyme molecules, and enzyme reactions. Researchers can leverage this wealth of data to annotate gene functions by linking genomic information with functional information.

For our functional annotation analysis, we employed the enrichGO and enrichKEGG functions available in the clusterProfiler package (Wu T. et al., 2021) within the R programming environment. These functions allowed us to perform GO and KEGG enrichment analysis on the genes within specific modules, aiding us in gaining valuable insights into the functional characteristics and pathways associated with these genes.

## 3 Results

### 3.1 Identification of co-expression gene modules

**Figure 5:**
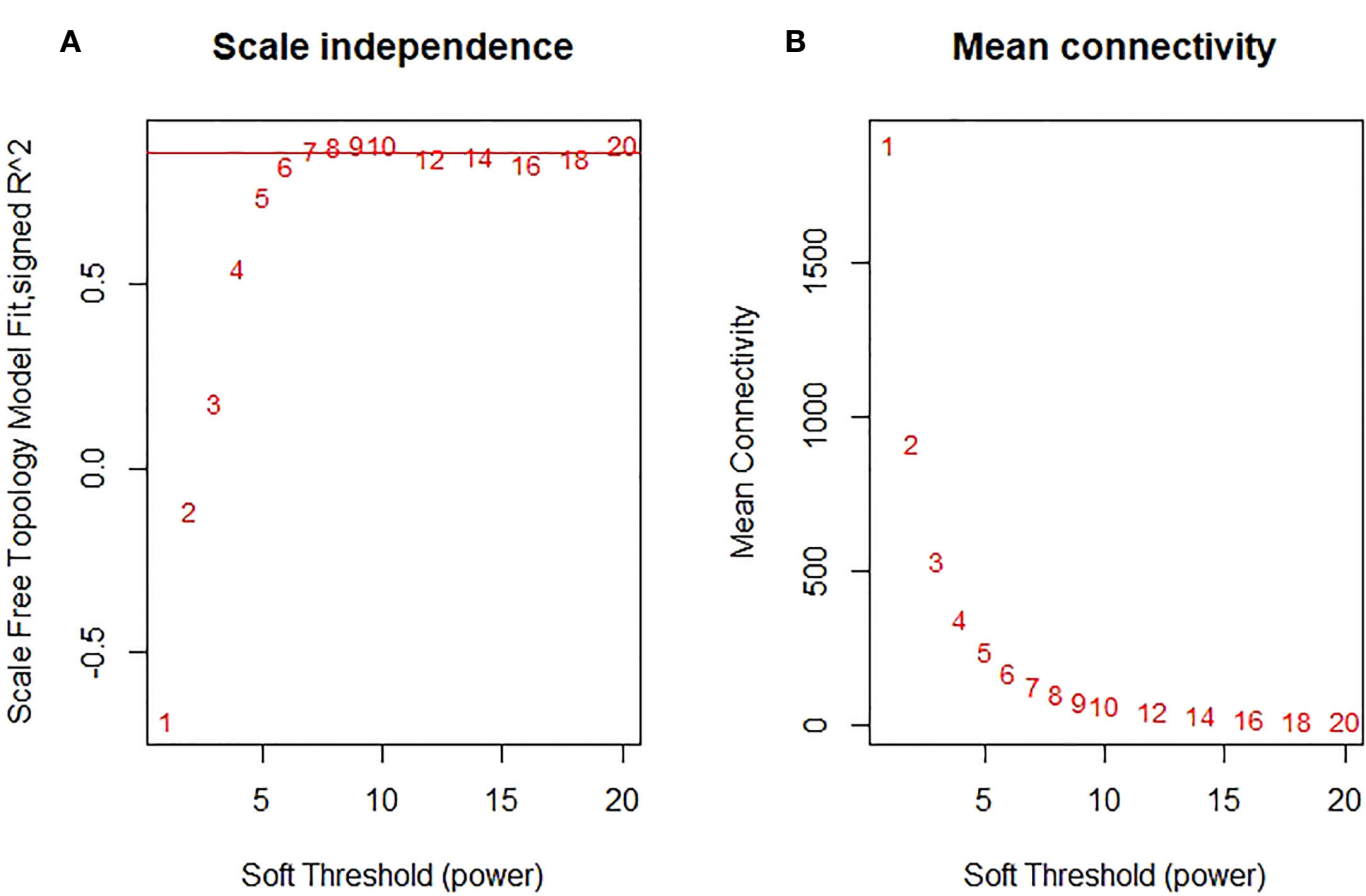
Analysis of network topology for various soft-thresholding powers. **(A)** The scalefree fit index (y-axis) as a function of the soft- thresholding power (x-axis). **(B)** the mean connectivity (degree, y-axis) as a function of the soft-thresholding power (x-axis).

In the curve fitting of the non-scale topology model with the soft threshold *β* and the fitting degree R^2^ (**Figure 5**), we observed that when R^2^ = 0.86, the gene connectivity curve tended to saturate. This observation indicates that the network conforms to the conditions of a non-scale distribution. The corresponding soft threshold at this point is determined to be *β*=7.

**Figure 6:**
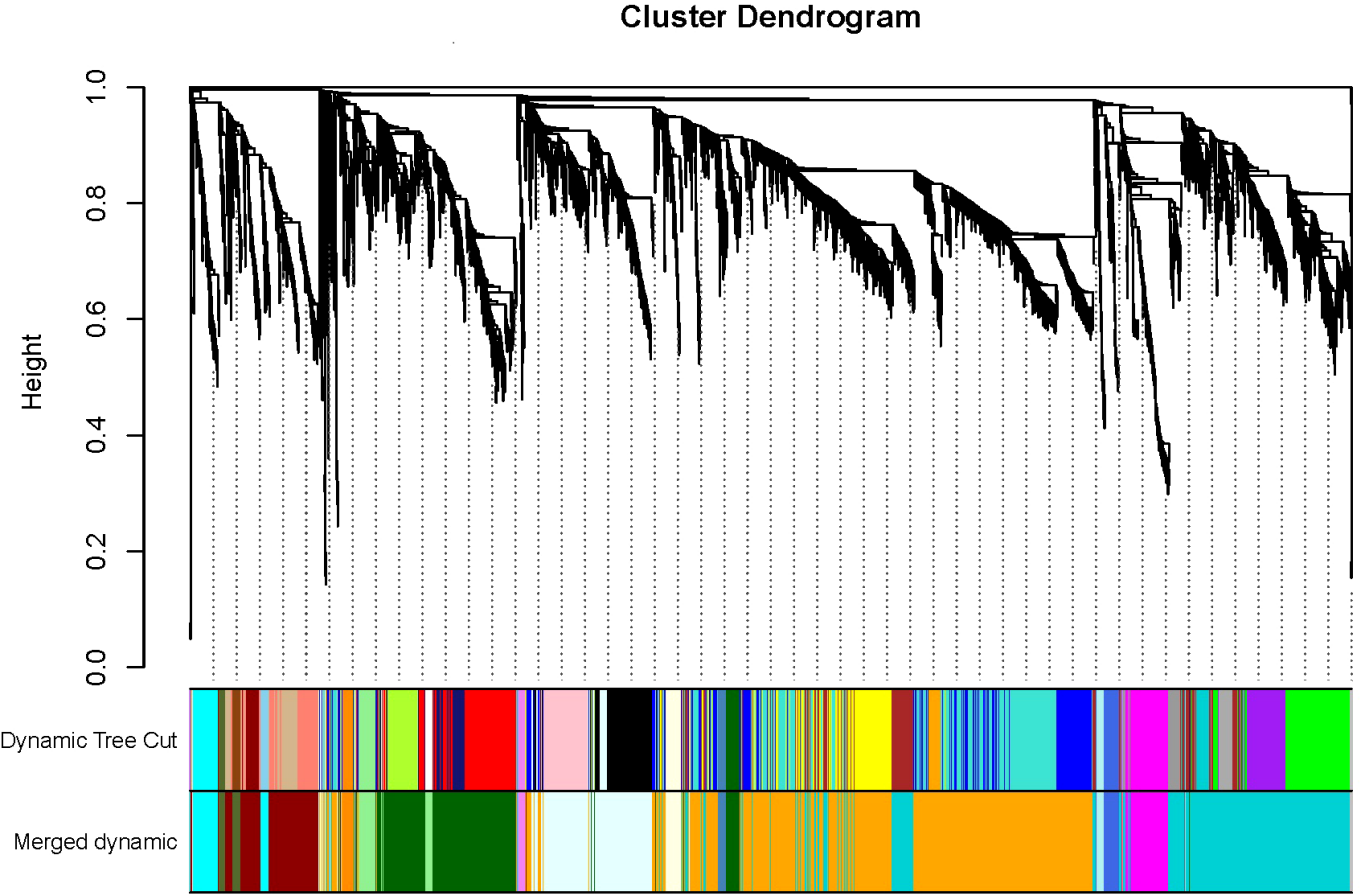
The co-expression genes clustering and the module cutting. Each branch represents a gene and each color below represents a gene co-expression module. The dynamic tree cut indicates the modules were divided based on the gene clustering results. The merged dynamic cut indicates the modules were divided by combining modules with similar expression patterns.

Subsequently, with the parameter β in place, hierarchical clustering was performed on the transcriptomics data obtained from 15 groups of transgenic and control lines. Gene modules were then formed through dynamic cutting, followed by the merging of similar modules. The outcome was the creation of 15 gene modules, excluding the grey module. To visually distinguish these modules, each module was randomly assigned a unique colour (**Tables S2**, **S3**). The figure depicting gene clustering and module cutting comprises two parts: the upper part visualizes the clustering dendrogram, demonstrating how genes are grouped based on expression pattern similarities, while the lower part illustrates the results of dynamic cutting for the modules and the subsequent outcome of module cutting after merging similar modules (**Figure 6**).

### 3.2 Correlation analysis of modules and traits

**Figure 7:**
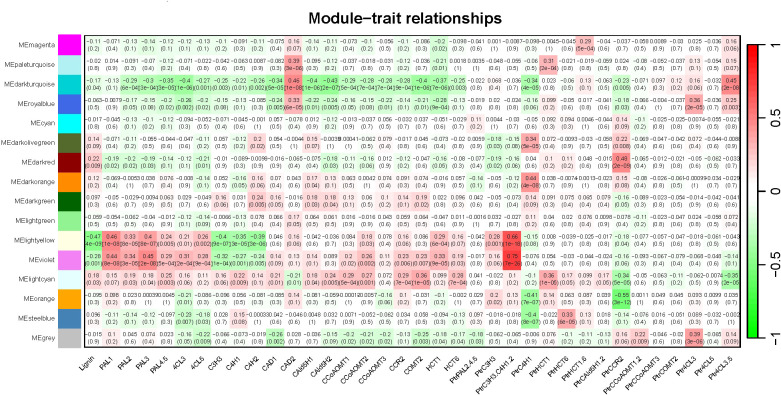
Heatmap of Module-trait relationships. The expression patterns of 15 modules (excluding the grey module) are shown by the heatmap. The module name is shown to the left side of each cell. Numbers in the table report the correlations of the corresponding module genes and trait, with the p-values (*) printed below the correlations in parentheses. Each column corresponds to a specific trait (from left To right: total lignin content, 20 pathway enzymes, and different treatments). The scale bar on the right indicates the range of possible correlations from positive (red) to negative (green).

We performed correlation analysis between all gene modules and traits (total lignin content, 20 pathway enzymes, and different treatments) to calculate the correlation coefficients (r). Subsequently, we generated a heatmap depicting the relationship between gene modules and traits (**Figure 7**). Our findings revealed that most monolignol genes (*PtrPAL5*, *PtrC3H3*, *PtrC4H1*, *PtrC4H2*, *Ptr4CL3*, *PtrCAD2*, *PtrHCT6*, *PtrCAld5H1*, and *PtrCAld5H2*) were clustered within the “MElightcyan” module. However, *PtrPAL2* and *PtrCAD2* were clustered within the “MEorange” and “MEroyalblue” modules, respectively.”

From the heatmap of the correlation analysis between modules and traits, we observed that the “MElightcyan” module, which contains most of the monolignol genes, exhibited a relatively low correlation with the total lignin content (|correlation coefficient| = 0.18), while the “MElightyellow” module showed the highest correlation with the total lignin content (|correlation coefficient| = 0.47), followed by the “MEviolet” module (|correlation coefficient| = 0.28). Furthermore, we found that the “MElightcyan” module, which includes most of the monolignol genes, exhibited a certain degree of correlation with the majority of pathway enzymes. The “MElightyellow” and “MEviolet” modules were primarily associated with pathway enzymes (PAL1, 4CL3, C3H3, C4H1, etc.) from the gene families of *PtrPAL*, *Ptr4CL*, *PtrC3H*, and *PtrC4H* monolignol genes. Additionally, we noticed that the treatment with triple gene knockdown of *PtrC3H3*, *PtrC4H1*, and *PtrC4H2* showed a strong correlation with these two modules, indicating that this treatment had the greatest impact on the total lignin content.

### 3.3 GO and KEGG enrichment analyses

**Figure 8:**
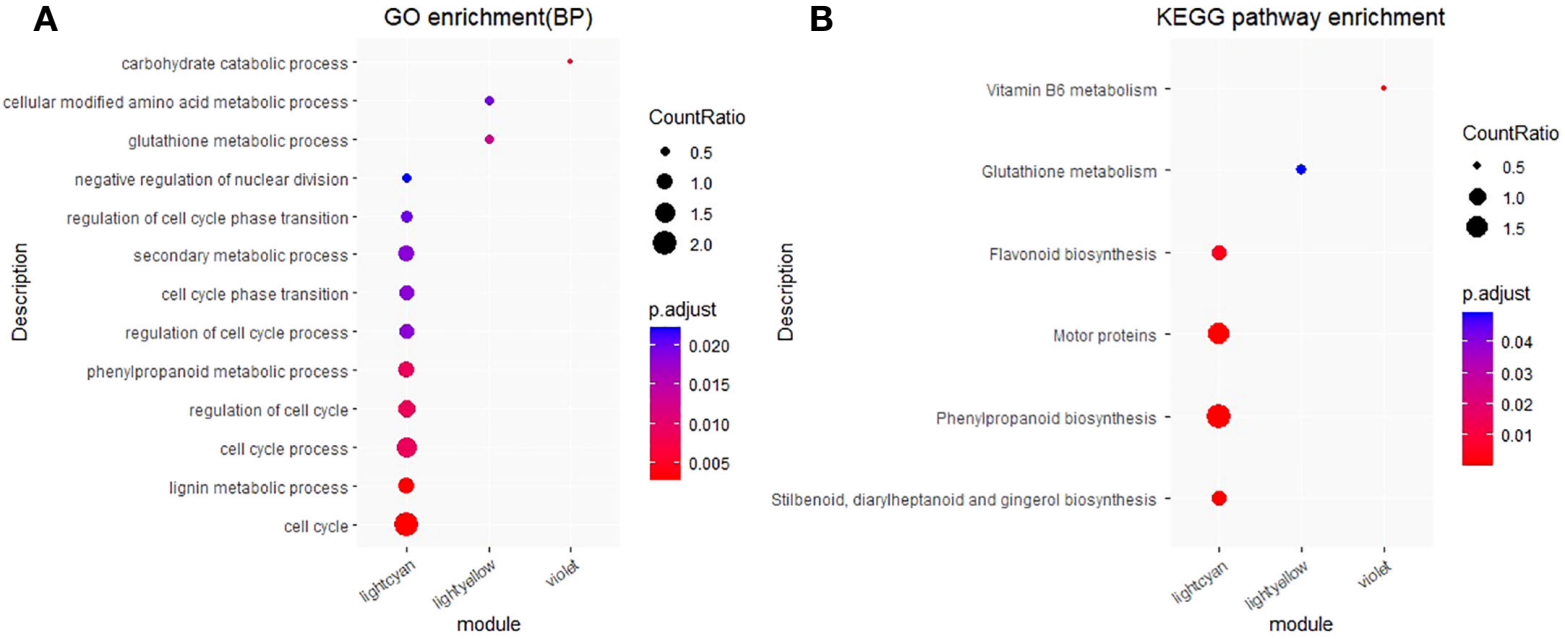
GO and KEGG enrichment analysis for the “MElightcyan”, “MElightyellow” and “MEviolet” gene modules. **(A)** GO and enrichment analysis. The top 10 biological process results of GO enrichment were selected to display. **(B)** KEGG and enrichment analysis. CountRatio: A score, the numerator is the number of genes enriched in this GO/KEGG entry and the denominator is the number of genes in specific module. p.adjust: corrected p value, the range for value of p.adjust from 0 (red) to 0.025 (blue). For the complete GO and KEGG enrichment results, please view **Tables S4** and **S5**.

We conducted GO and KEGG enrichment analyses for the “MElightcyan”, “MElightyellow” and “MEviolet” gene modules. The top 10 results of the GO (focusing on biological processes) and KEGG enrichment analysis results for each module, are shown in **Figure 8** (**Tables S4**, **S5**).

The “MElightcyan” module, comprising most of the monolignol genes, reflected significant enrichment (P< 0.05) of pathways such as “cell cycle”, “lignin metabolic process”, “phenylpropanoid metabolic process”, and “secondary metabolic process”. Additionally, this module indicated enrichment of pathways such as “flavonoid biosynthesis” and “phenylpropanoid biosynthesis”. In contrast, the “MElightyellow” module, displaying the highest correlation with the total lignin content, indicated significant enrichment in pathways related to “glutathione metabolic process” and “cellular modified amino acid metabolic process,” as well as in the “glutathione metabolism” pathway. The “MEviolet” module reflected significant enrichment in the “carbohydrate catabolic process” and “vitamin B6 metabolism” pathways. The GO and KEGG enrichment results for these three modules, which awere closely associated with lignin, indicate that the total lignin content is influenced not only by genes involved in the “lignin biosynthesis”, “phenylpropanoid biosynthesis” and “flavonoid biosynthesis” pathways but also by genes involved in the “glutathione metabolic process”, “cellular modified amino acid metabolic process” and “carbohydrate catabolic process” pathways.

### 3.4 Correlation analysis and visualization of the genes co-expression network

We conducted a detailed analysis of the genes within the “MElightyellow” module, which demonstrated the highest correlation with the total lignin content. As a result, we identified eight types of genes present in this module, including cytochrome P450 genes, cinnamyl-alcohol dehydrogenase genes, UDP-glucosyltransferase genes, UDP-glycosyltransferase superfamily protein genes, glutathione-S-transferase genes, wall-associated kinase genes, MATE efflux family protein genes, and indole-3-acetate beta-D-glucosyltransferase genes.

**Figure 9:**
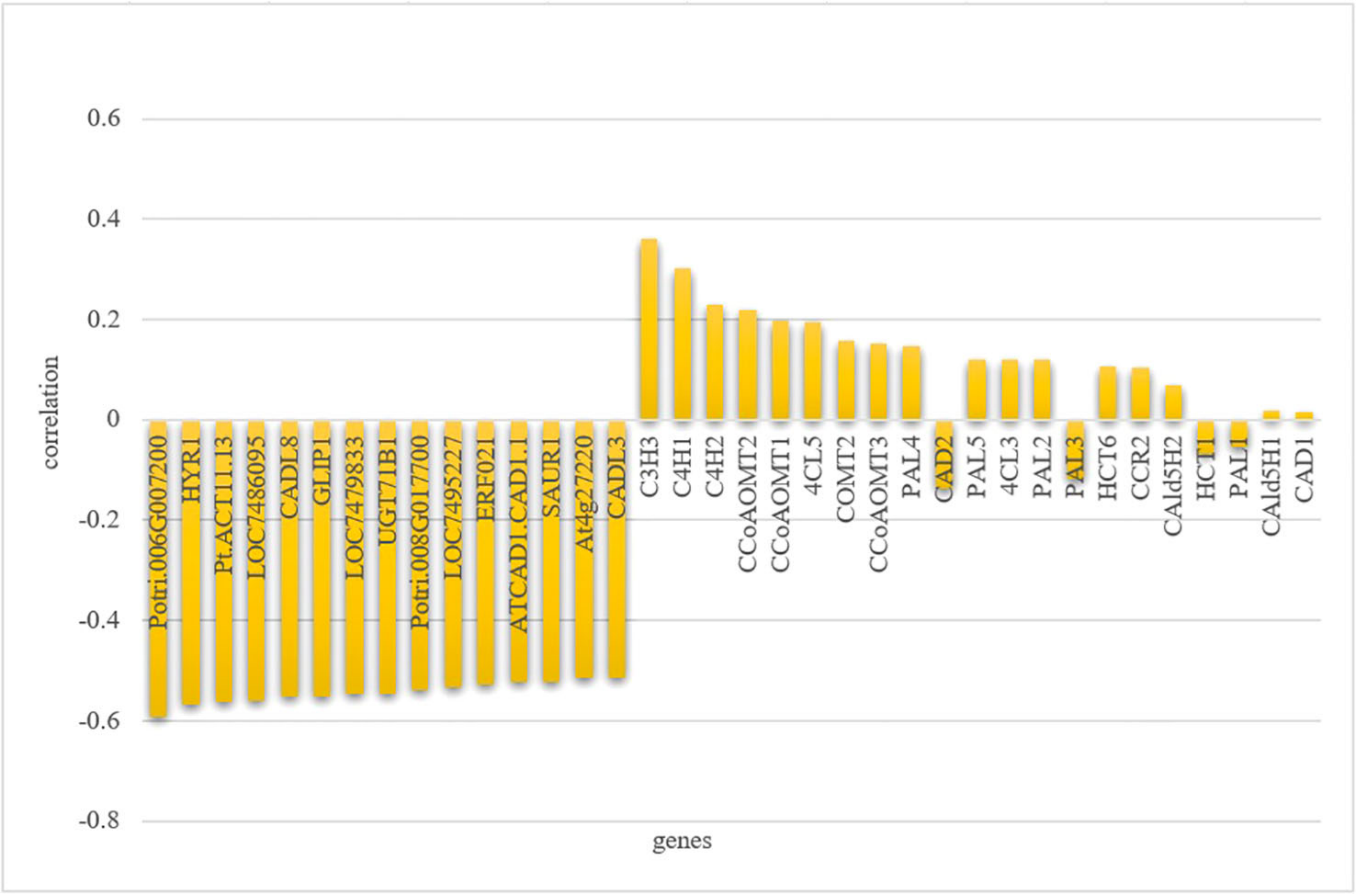
Correlation analysis of gene expression levels with total lignin content. The genes, including the top 15 genes displaying the strongest correlation with total lignin content, as well as monolignol genes, is shown on the x-axis; The correlation coefficient of genes and total lignin content is shown on the y-axis.

Next, to compare the correlation levels between the genes from the “MElightyellow” module that most correlated with the total lignin content, as well as monolignol genes and the total lignin content, we conducted a correlation analysis using the R package cor (**Figure 9**; **Table S6**). It is worth noting that we found that the correlation (highest: |r| = 0.59, lowest: |r| = 0.52) between the top 15 genes in the “MElightyellow” module and the lignin content was much higher than that between monolignol genes and the lignin content (highest: |r| = 0.36, lowest: |r| = 0.02). We have systematically compiled information about the top 15 genes with the strongest correlation to the total lignin in the “MElightyellow” module, as outlined in **Table 1**. The kME (eigengene connectivity) serves as intermediate data during the analysis of gene co-expression networks (**Table S7**), and the methodology for calculating kME is detailed in Section 2.4. Specifically, the higher the kME value, the stronger the degree of gene connectivity, indicating greater significance and centrality within the network. “|r|” represents the absolute value of the correlation coefficient.

**Table 1:** The top 15 genes displaying the strongest correlation with total lignin content.

Locus	Symbol	kME	|r|	Reference
*Potri.006G007200*	*Potri.006G007200*	0.979	0.591	\
*Potri.006G007100*	*HYR1*	0.947	0.567	(Xu et al., 2019)
*Potri.008G099100*	*Pt.ACT11.13*	0.869	0.563	(Park et al., 2020)
*Potri.007G107600*	*LOC7486095*	0.881	0.559	\
*Potri.006G199100*	*CADL8*	0.980	0.553	(Kim et al., 2007; Chao et al., 2022; Lee et al., 2022)
*Potri.004G084400*	*GLIP1*	0.856	0.553	(Han et al., 2019; Miao et al., 2019)
*Potri.007G147000*	*LOC7479833*	0.792	0.547	\
*Potri.016G017200*	*UGT71B1*	0.946	0.546	(Jiang, 2022)
*Potri.008G017700*	*Potri.008G017700*	0.875	0.540	\
*Potri.014G123500*	*LOC7495227*	0.833	0.533	\
*Potri.013G101100*	*ERF021*	0.819	0.528	(Zeng et al., 2020)
*Potri.T149600*	*CAD1*	0.922	0.523	(Chao et al., 2022; Lee et al., 2022)
*Potri.003G167400*	*SAUR1*	0.900	0.522	(Tong et al., 2022)
*Potri.019G069200*	*At4g27220*	0.879	0.516	(Cortaga et al., 2022)
*Potri.016G023300*	*CADL3*	0.920	0.515	(Kim et al., 2007; Lee et al., 2022)

**Figure 10:**
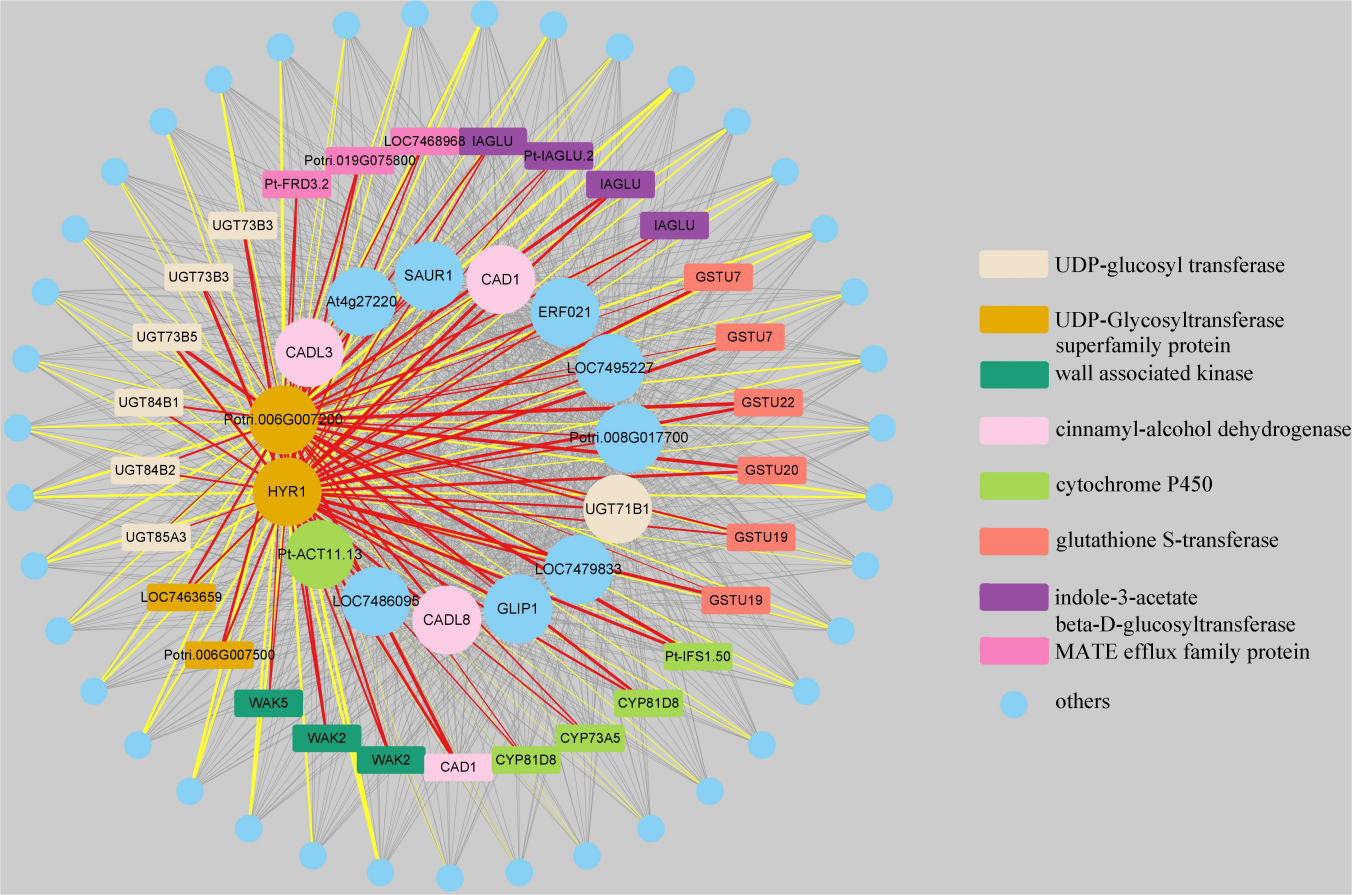
Visualization of weighted genes co-expression network in the “MElightyellow” module. The 15 genes with the strongest correlation to the total lignin content were selected as central nodes, while the eight types of genes were represented by different colors, and genes not belonging to these types were represented in blue. The nodes represent genes, and the edges represent co-expression relationships between genes, with edge thickness indicating the strength of the co-expression relationship. The edges in red and yellow respectively represent the relationships between two UDP-glycosyltransferase superfamily protein genes and eight types of genes, as well as the co-expression relationships between two UDP-glycosyltransferase superfamily protein genes and other genes beyond these eight types. For the complete genes coexpression network results, please view **Table S8**.

Finally, we utilized Cytoscape 3.9.1 software to visualize the co-expression network constructed from genes in the “MElightyellow” module that showed the highest correlation with the total lignin content (**Figure 10**; **Table S8**). The 15 genes with the strongest correlation to the total lignin content were selected as central nodes, while the eight types of genes were represented by different colours, and genes not belonging to these types were represented in blue. In the network, nodes represent genes, and the edges represent co-expression relationships between genes, with edge thickness indicating the strength of the co-expression relationship. Specifically, the edges in red and yellow represent the relationships between two UDP-glycosyltransferase superfamily protein genes and eight types of genes, respectively, as well as the co-expression relationships between two UDP-glycosyltransferase superfamily protein genes and other genes beyond these eight types. Notably, we observed that these 15 genes, which had the highest correlation with the total lignin content, exhibited co-expression relationships with nearly all genes within the “MElightyellow” module.

## 4 Conclusions and discussion

Firstly, we performed weighted gene co-expression network analysis (WGCNA) on a dataset containing 5894 differentially expressed genes from 139 samples. Our analysis revealed that enzymes from the *PtrPAL*, *Ptr4CL*, *PtrC3H*, and *PtrC4H* gene families may have a closer relationship with the total lignin content(Kim et al., 2023; Wu et al., 2023). In addition, the co-downregulation of three genes, *PtrC3H3*, *PtrC4H1*, and *PtrC4H2*, had the greatest impact on total lignin content, which is consistent with the finding reported by Kim et al. (Kim et al., 2020).

Secondly, by performing GO and KEGG analyses on the lignin-related modules, we discovered that the total lignin content is influenced not only by genes involved in the “lignin biosynthesis”, “phenylpropanoid biosynthesis” and “flavonoid biosynthesis” pathways but also by genes involved in the “glutathione metabolic process”, “cellular modified amino acid metabolic process” and “carbohydrate catabolic process” pathways. Moreover, glutathione peroxidase (GSH-PX) is one of the peroxidases, involved in reactive oxygen species metabolism and lignin metabolism. It can significantly influence the content of total phenols, which are precursors of lignin synthesis, and the phenylalanine ammonia-lyase involved in monolignol biosynthesis, thereby influencing the total lignin content (Yu et al., 2011).

Finally, further analysis of the genes within the “MElightyellow” module, which showed the highest correlation with the total lignin content, revealed that this module mainly contains eight types of genes. Among the top 15 genes with the highest correlation to the total lignin content and expression levels of all genes within the module, four types of genes were identified: 1. three cinnamyl-alcohol dehydrogenase genes, namely *CAD1*, *CADL3*, and *CADL8* (Kim et al., 2007; Chao et al., 2022; Lee et al., 2022); 2. two UDP-glycosyltransferase superfamily protein genes, *Potri.006G007200* and *HYR1*, showed the closest correlation with the total lignin content, with *HYR1* found to be significantly downregulated after exposure to coumarin (Xu et al., 2019); 3. one UDP-glucosyl transferase gene, *UGT71B1*, was reported to play a critical role in coumarin metabolism and glycosylation in regulating the efficacy of secondary metabolites (Jiang, 2022); and 4. one cytochrome P450 gene, *Pt-ACT11.13* (Park et al., 2020). Importantly, among these 15 genes, there are several noteworthy ones. The *GLIP1* gene involved in pathogen defence and is regulated by the transcription factor WRKY (Han et al., 2019; Miao et al., 2019), the *ERF021* gene directly regulates the expression of monolignol genes (Zeng et al., 2020), the *SAUR1* gene is an auxin-inducible gene that promotes plant cell division and elongation (Tong et al., 2022), and the *At4g27220* gene functions as a resistance protein (Cortaga et al., 2022). However, genes *LOC7486095*, *LOC7479833*, *LOC7495227*, and *Potri.008G017700* have not been previously reported in relation to lignin.

Therefore, we can infer that the monolignol genes *PtrC3H3*, *PtrC4H1*, and *PtrC4H2*, which belong to the cytochrome P450 gene type, may have a significant impact on the total lignin content. Among the top 15 genes with the highest correlation to the total lignin content, three cinnamyl-alcohol dehydrogenase genes were present. This gene type is involved in the final step of the monolignol biosynthetic pathway, suggesting that it may significantly influence the total lignin content. Furthermore, the UDP-glycosyltransferase superfamily protein genes and UDP-glucosyl transferase genes are closely related to coumarin, indicating that they may influence the total lignin content by influencing coumarin metabolism.

However, despite identifying genes that may influence the total lignin content, the correlation between gene modules and total lignin content did not reach the expected level. This lower correlation could be attributed to the fact that the knockdown of monolignol genes did not entirely reduce the total lignin content to zero. Additionally, the differences between transgenic and control lines are often smaller compared to lines under different stress conditions, and these relatively insignificant differences may affect the degree of correlation between modules and traits.

In conclusion, for a single species (*P. trichocarpa*), utilizing a combination of t-test, differential gene expression analysis, correlation analysis, and WGCNA on multi-omics data, including data related to the transcriptome, proteome, and total lignin content of multiple transgenic lines, allows us to establish a connection between gene expression levels and total lignin content. This approach effectively revealed information about genes that influence total lignin content and facilitates a better understanding of the impact of genes on lignin. Therefore, in future lignin research, we should not only focus on studying genes related to the monolignol biosynthetic pathway and their regulatory factors but also investigate which genes or pathways may have an impact on lignin content. By considering the overall metabolic activities of organisms and utilizing advanced analysis methods, a more comprehensive exploration of lignin biosynthesis can be achieved, ultimately leading to the strategic development and utilization of lignin. This study conducted an initial exploration of gene regulation on total lignin content. In the future, more advanced techniques, such as machine learning and gene inference networks, are expected to be applied to more in-depth research in this field.

## Data availability statement

Publicly available datasets were analyzed in this study. This data can be found here: The transcriptome are available under GEO accession number GSE78953 [https://www.ncbi.nlm.nih.gov/geo/query/acc.cgi?acc=GSE78953], while the data sets of proteo- mics and total lignin content are available on CyVerse [https://datacommons.cyverse.org/browse/iplant/home/shared/Lignin SystemsDB]. The annotation information of all genes was acquired from two databases, JGI [https://jgi.doe.gov/] and NCBI [https://www.ncbi.nlm.nih.gov/].

## Author contributions

JZ, AW, and KC developed the concept of the article. JZ analyzed and wrote the paper. AW provided guidance on technical analysis methods. All authors contributed to the article and approved the submitted version.
